# Combined discriminative value of SII and sdLDL-C for 1-year major adverse cardiovascular events in patients with unstable angina pectoris

**DOI:** 10.3389/fcvm.2026.1840437

**Published:** 2026-08-07

**Authors:** Qian Zhao, Liang Peng, Lei Li

**Affiliations:** 1 Department of Cardiovascular Medicine, Central Hospital Affiliated to Shandong First Medical University, Jinan Central Hospital, Jinan, China; 2 Electrocardiogram (ECG) Room, Special Examination Department, Jinan Eighth People's Hospital, Jinan, China; 3 Henan Medical University, Xinxiang, China

**Keywords:** major adverse cardiovascular events, risk stratification, small dense low-density lipoprotein cholesterol, systemic immune-inflammation index, unstable angina pectoris

## Abstract

**Objective:**

To assess the associations of the systemic immune-inflammation index (SII) and small dense low-density lipoprotein cholesterol (sdLDL-C) with 1-year major adverse cardiovascular events (MACE) in patients with unstable angina pectoris (UAP) and to evaluate their individual and combined discriminative performance.

**Methods:**

This single-center retrospective cohort study included 157 patients diagnosed with UAP who underwent coronary angiography between January 2022 and June 2024. Routine blood counts, lipid profiles, and electrocardiography were obtained within 24 h after admission. SII was calculated as neutrophil count  ×  platelet count/lymphocyte count. The prespecified endpoint was the occurrence of at least one MACE during the fixed 1-year follow-up period, including cardiac death, nonfatal myocardial infarction, target vessel revascularization, or rehospitalization for worsening heart failure. Patients were classified into the MACE group (*n* = 38) and the non-MACE group (*n* = 119). Variables showing statistical significance in the univariable analyses were entered into a multivariable logistic regression model to identify factors independently associated with 1-year MACE. Receiver operating characteristic curves were used to assess the discriminative performance of SII, sdLDL-C, and their combination. Internal validation was performed using 1,000 bootstrap resamples.

**Results:**

Compared with the non-MACE group, patients in the MACE group were older, had a higher prevalence of diabetes mellitus, higher Gensini scores, and more frequent multi-lead ischemic changes on electrocardiography. The MACE group also had higher WBC, NEUT, PLT, SII, TG, and sdLDL-C levels and lower LYM levels (all *P* < 0.05). Multivariable logistic regression showed that SII ≥ 1,100 (OR = 3.58, 95% CI: 1.79–7.14; *P* < 0.001), sdLDL-C ≥ 1.02 mmol/L (OR = 2.91, 95% CI: 1.42–5.97; *P* = 0.003), Gensini score ≥ 45 points (OR = 2.67, 95% CI: 1.31–5.46; *P* = 0.006), and multi-lead ischemic changes (OR = 2.43, 95% CI: 1.18–5.01; *P* = 0.017) were independently associated with 1-year MACE. The AUCs of SII, sdLDL-C, and the combined SII–sdLDL-C model were 0.832, 0.789, and 0.914, respectively. After bootstrap validation, the optimism-corrected AUC of the combined model was 0.893, with a calibration slope of 0.87.

**Conclusion:**

Elevated SII and sdLDL-C levels were independently associated with MACE within 1 year in patients with UAP. Their combination showed better discrimination than either marker alone. However, because of the limited number of patients with MACE and the absence of external validation, these findings should be considered exploratory and require confirmation in larger prospective multicenter cohorts.

## 1 Introduction

Coronary heart disease (CHD) remains a leading cause of morbidity and mortality worldwide (1). Unstable angina pectoris (UAP), a clinical presentation within acute coronary syndrome, is characterized by new-onset, worsening, or rest angina without evidence of acute myocardial necrosis (2, 3). Although advances in cardiac troponin testing have changed the contemporary diagnosis of UAP, affected patients remain at risk of myocardial infarction, cardiac death, and recurrent ischemic events. Therefore, early identification of high-risk patients and accurate risk stratification remain clinically important.

Recent studies (4, 5) have shown that inflammatory responses play a key role in the occurrence and progression of atherosclerosis as well as plaque instability. Infiltration of inflammatory cells can promote endothelial dysfunction, lipid deposition, and thinning of the fibrous cap, thereby increasing the risk of plaque rupture. The systemic immune-inflammation index (SII) is a comprehensive inflammatory indicator calculated based on peripheral blood neutrophil count (NEUT), lymphocyte count (LYM), and platelet count (PLT). It simultaneously reflects the inflammatory status and immune balance of the body. Compared with a single inflammatory parameter, SII integrates multiple inflammatory and thrombosis-related factors and is considered a relatively stable indicator for evaluating the intensity of systemic inflammatory responses (6). Previous studies (7) have shown that elevated SII levels are closely associated with the severity of coronary artery disease and increased risk of acute coronary events, and it may serve as a potential predictor of prognosis in cardiovascular diseases. However, evidence regarding the association between SII and 1-year major adverse cardiovascular events (MACE) in patients with UAP remains limited.

In addition to inflammatory factors, abnormalities in lipid metabolism are one of the core mechanisms underlying the development of CHD. Low-density lipoprotein cholesterol (LDL-C) has long been recognized as a major risk factor for atherosclerosis. However, increasing evidence (8, 9) indicates that small dense low-density lipoprotein cholesterol (sdLDL-C) has stronger atherogenic properties than normal LDL particles. sdLDL-C particles are smaller and denser, making them more likely to penetrate the vascular endothelium and become retained in the vascular wall. They are also more prone to oxidative modification, which enhances their pro-inflammatory, pro-thrombotic, and endothelial-damaging effects. Previous studies have associated elevated sdLDL-C levels with an increased risk of acute coronary syndrome (10); however, the value of sdLDL-C for assessing 1-year clinical outcomes in patients with UAP remains unclear.

Inflammation and lipid abnormalities interact during the development and progression of atherosclerosis (11, 12). Combining biomarkers that reflect these two clinical domains may provide more comprehensive risk information than either marker alone. However, evidence regarding the combined discriminative value of SII and sdLDL-C in patients with UAP remains limited. Therefore, this study evaluated the associations of SII and sdLDL-C with MACE during a fixed 1-year follow-up period and assessed their individual and combined discriminative performance.

## 2 Materials and methods

### 2.1 Study design, subjects, and ethics

**Figure 1:**
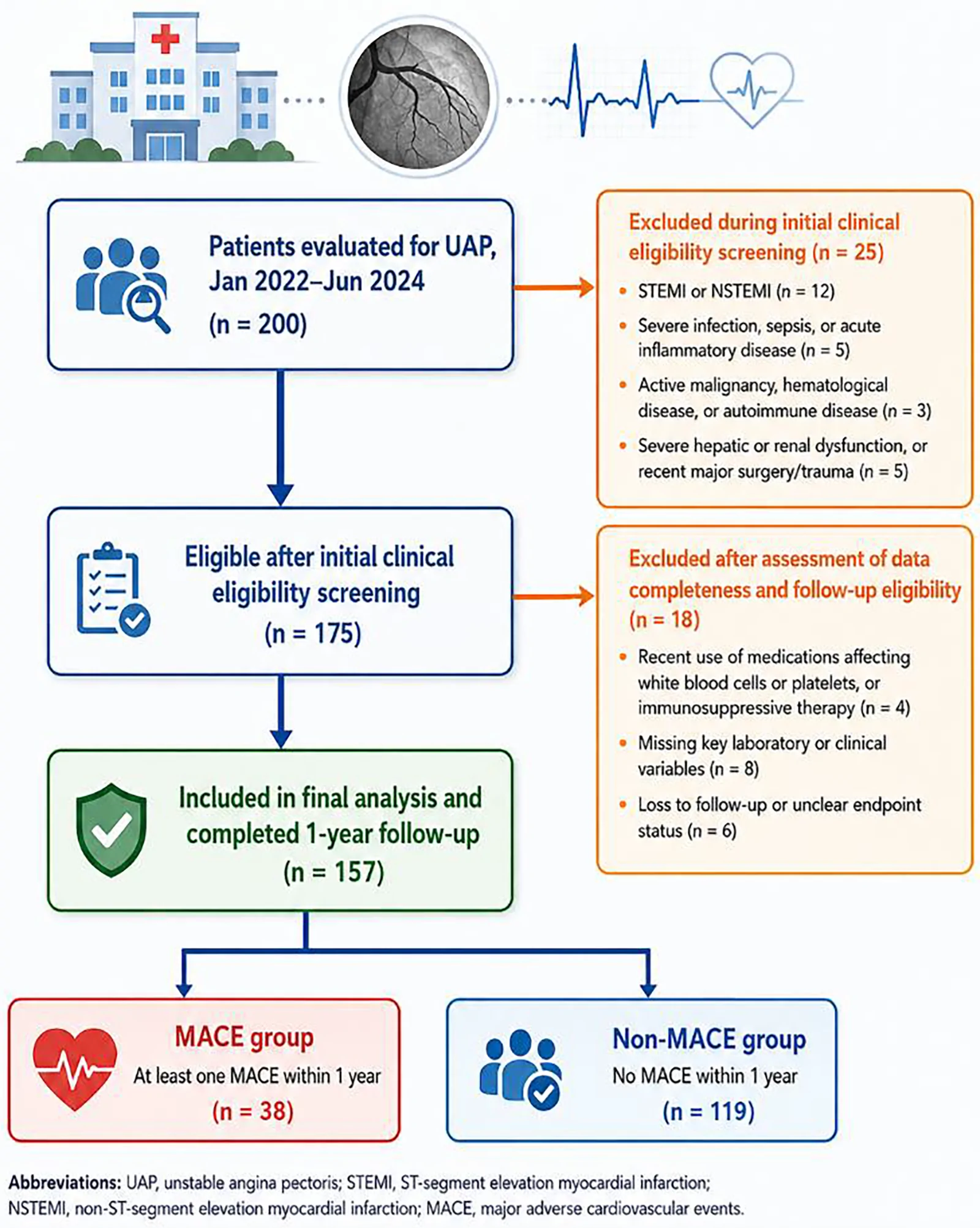
Flowchart of participant screening, exclusion, enrollment, follow-up, and grouping. UAP, unstable angina pectoris; STEMI, ST-segment elevation myocardial infarction; NSTEMI, non-ST-segment elevation myocardial infarction; MACE, major adverse cardiovascular events.

This was a single-center retrospective cohort study. The participant screening, exclusion, enrollment, follow-up, and grouping process is presented in Figure 1. A total of 200 consecutive patients evaluated for UAP in the Department of Cardiology of our hospital between January 2022 and June 2024 were screened for eligibility. Of these, 43 patients were excluded according to the prespecified exclusion criteria, data completeness, and follow-up eligibility. Finally, 157 patients with angiographically confirmed UAP who completed the 1-year follow-up were included in the analysis. According to whether at least one MACE occurred during the fixed 1-year follow-up period, the patients were divided into the MACE group (*n* = 38) and the non-MACE group (*n* = 119). The study protocol was reviewed and approved by the Medical Ethics Committee of our hospital (approval number: XNLC2023ZBJC-01). Because this was a retrospective study and all data were derived from existing clinical records, the ethics committee approved the waiver of written informed consent. The study procedures complied with the Declaration of Helsinki and relevant medical ethics guidelines. All patient data were de-identified before analysis to protect confidentiality.

### 2.2 Inclusion and exclusion criteria

(1) Inclusion criteria: 1) age ≥18 years, regardless of sex; 2) meeting the clinical diagnostic criteria for UAP and having undergone coronary angiography; 3) completion of routine blood tests and lipid-related examinations within 24 h after admission, enabling acquisition of SII and sdLDL-C results; 4) complete clinical data with the ability to complete at least 1 year of follow-up and a clearly defined MACE outcome.
(2) Exclusion criteria: 1) diagnosis of acute ST-segment elevation myocardial infarction or non-ST-segment elevation myocardial infarction based on clinical manifestations, electrocardiographic findings, and serial hs-cTnI measurements, as described in Section 2.3.1; 2) presence of severe infection, sepsis, or acute inflammatory disease; 3) presence of active malignancy, hematological disease, or autoimmune disease; 4) severe hepatic or renal dysfunction or recent major surgery/trauma; 5) recent (within 4 weeks before admission) use of medications affecting white blood cells or platelets, or receipt of immunosuppressive therapy that could not be adequately evaluated; 6) missing key clinical or laboratory variables required for the primary analyses, or loss to follow-up or insufficient follow-up information preventing reliable determination of the 1-year MACE endpoint.

### 2.3 Diagnostic criteria and definitions

#### 2.3.1 Diagnostic criteria for UAP

The diagnosis of UAP was established according to relevant guidelines (13) based on ischemic symptoms, electrocardiographic findings, serial high-sensitivity cardiac troponin I (hs-cTnI) measurements, and coronary angiography. The clinical criteria included new-onset or worsening ischemic chest pain at rest or during mild exertion, increased frequency or prolonged duration of chest pain episodes, or reduced response to nitrates. hs-cTnI was measured at admission and repeated 3 h after the initial measurement. An additional measurement was performed at 6 h when acute myocardial infarction remained clinically suspected. NSTEMI was excluded when serial hs-cTnI measurements did not fulfill the diagnostic criteria for acute myocardial infarction, namely a rise and/or fall in cardiac troponin with at least one value above the assay-specific 99th-percentile upper reference limit together with clinical evidence of myocardial ischemia. Coronary angiography showed atherosclerotic stenosis or plaque changes in at least one major coronary artery that were consistent with the clinical presentation.

#### 2.3.2 Follow-up and definition of MACE endpoint events

Patients were followed for 1 year after discharge through outpatient record review, inpatient medical record retrieval, and telephone contact. The prespecified endpoint was the occurrence of at least one MACE during this fixed follow-up period. Because the exact date of the first MACE was not consistently available in the retrospective records, the endpoint was analyzed as a binary outcome rather than a time-to-event outcome. MACE comprised cardiac death, nonfatal myocardial infarction, target vessel revascularization [including percutaneous coronary intervention (PCI) or coronary artery bypass grafting (CABG)], and rehospitalization for worsening heart failure. The individual components of MACE were also summarized descriptively. Because a patient could experience more than one component during follow-up, the component counts were not mutually exclusive, and the corresponding percentages were calculated using the 38 patients with MACE as the denominator. Each endpoint event was determined based on comprehensive evaluation of hospitalization records, discharge summaries, readmission records, outpatient follow-up records, and, when necessary, laboratory or imaging examination results. If patients received treatment at other hospitals, verification was performed by obtaining relevant discharge records, examination reports, or written documents provided by the patients or their family members. In cases where the determination of endpoint events was controversial, two investigators independently reviewed the data; if disagreement occurred, a third investigator participated in the discussion to make the final decision.

#### 2.3.3 Definition of electrocardiographic ischemic changes

Ischemic changes on the admission 12-lead electrocardiogram were defined as horizontal or downsloping ST-segment depression of ≥ 0.05 mV in at least two contiguous leads, or T-wave inversion or flattening in at least two contiguous leads consistent with myocardial ischemia. These findings were defined as “multi-lead ischemic changes.”

#### 2.3.4 Severity of coronary artery lesions (gensini score)

The Gensini score (14) was calculated for all patients based on coronary angiography results to quantify the degree of coronary artery stenosis and the extent of lesions. The score was calculated by qualified interventional cardiologists according to angiography reports and images. In case of disagreement, another senior physician reviewed the results and a consensus was reached. The Gensini score was used as an indicator of coronary lesion burden in the statistical analysis.

### 2.4 Data collection and variable definitions

#### 2.4.1 General information and medical history

Demographic and clinical variables were retrospectively collected from the electronic medical records, including sex, age, body mass index (BMI), hypertension, diabetes mellitus, stroke, smoking history, and previous PCI history.

#### 2.4.2 Medication and treatment information

Treatment information during the index hospitalization and at discharge was retrospectively collected from the electronic medical records. The revascularization strategy during the index hospitalization was categorized as medical therapy alone, PCI, or CABG. Major discharge medications included dual antiplatelet therapy, statins, *β*-blockers, ACEI/ARB/ARNI, and nitrates. High-intensity statin therapy was defined as atorvastatin 40–80 mg/day or rosuvastatin 20–40 mg/day; other statin regimens were classified as moderate- or low-intensity therapy. Because medication adherence and treatment changes during follow-up were not consistently documented in the retrospective records, long-term adherence could not be reliably quantified.

#### 2.4.3 Laboratory examinations

Venous blood samples were collected from all patients within 24 h after admission. The testing items included: (1) routine blood tests: white blood cell count (WBC), neutrophil count (*N*EUT), lymphocyte count (LYM), platelet count (PLT), etc.; (2) lipid profile: total cholesterol (TC), triglycerides (TG), high-density lipoprotein cholesterol (HDL-C), and low-density lipoprotein cholesterol (LDL-C); (3) sdLDL-C was measured in the central laboratory of our hospital using a direct homogeneous clearance assay with a commercially available reagent kit obtained from Zhejiang Dongou Diagnostic Products Co., Ltd., China. All measurements were performed according to the manufacturer's instructions and the laboratory's standardized operating procedures. Only results generated by the hospital laboratory using this standardized assay were included, and results obtained using alternative methods or from external laboratories were not combined. Routine instrument calibration and internal quality control were performed according to the laboratory quality-management procedures, and patient results were accepted only when the quality-control criteria were met. To reduce variation related to the timing of measurement, the first valid sdLDL-C result obtained from venous blood collected within 24 h after admission was used. If multiple measurements were available during this period, the earliest valid result was included in the analysis.

#### 2.4.4 Calculation method of SII

SII was calculated according to the formula: SII=NEUT (×10⁹/L) × PLT (×10⁹/L)/LYM (×10⁹/L). SII was described as a continuous variable and was further stratified according to the optimal cutoff value determined by ROC analysis.

### 2.5 Quality control and reproducibility assurance

All laboratory procedures were performed by trained and qualified personnel in accordance with standardized operating procedures. Instruments were calibrated and subjected to performance and quality-control checks according to the manufacturers' recommendations and the laboratory's quality-management schedule. For sdLDL-C measurement, assay calibration and internal quality-control results were reviewed before patient results were accepted; if the predefined quality-control criteria were not met, the cause was investigated and the assay was repeated when necessary.

Study data were independently entered into an electronic database by two researchers and cross-checked for accuracy. Potentially missing or inconsistent data were verified against the original electronic medical records. Patients were excluded before analysis if key variables required for the primary analyses remained unavailable or if the 1-year MACE endpoint could not be reliably determined. Eight patients were excluded because of missing key clinical or laboratory variables, and six were excluded because of loss to follow-up or an indeterminate endpoint status. The final cohort of 157 patients had complete data for all variables included in the primary analyses; therefore, complete-case analysis was performed, and no statistical imputation was used. All study data were de-identified and stored in a password-protected database accessible only to authorized study personnel.

### 2.6 Statistical analysis

Data analysis was performed using GraphPad Prism 9.0 (GraphPad Software, USA) and R software version 4.3.2. Continuous variables were first tested for normality using the Shapiro–Wilk test. Normally distributed data were expressed as mean ± standard deviation and compared between groups using the independent-samples t-test. Non-normally distributed data were expressed as median and interquartile range and analyzed using the Mann–Whitney U test. Categorical variables were expressed as number and percentage, and between-group comparisons were performed using the *χ*^2^ test or Fisher's exact test, as appropriate. Univariate analyses were initially performed to identify factors associated with MACE. Given the limited number of MACE events, the candidate predictor set was restricted to the seven variables that were statistically significant in the univariate analyses: age, diabetes mellitus, TG, Gensini score, multi-lead ischemic changes, SII, and sdLDL-C. These variables were entered into the multivariable logistic regression model to identify factors independently associated with MACE. Logistic regression was selected because the primary outcome was defined as the occurrence or non-occurrence of MACE within a fixed 1-year period. Cox proportional hazards regression was not performed because the exact time to the first event was not consistently available for all participants. ROC curves were used to evaluate the discriminative performance of SII, sdLDL-C, and their combination. The combined SII–sdLDL-C model was constructed by simultaneously entering the continuous values of SII and sdLDL-C into a binary logistic regression model, and the resulting predicted probabilities were used for ROC analysis. The AUC, 95% confidence interval, sensitivity, specificity, and Youden index were calculated. The optimal cutoff value for each individual marker was defined as the value that maximized the Youden index in the present cohort. Differences between AUCs were compared using the DeLong test. To evaluate model stability and potential overfitting, internal validation was performed using 1,000 bootstrap resamples. The complete model-building procedure was repeated in each bootstrap sample, and the apparent AUC, bootstrap optimism, optimism-corrected AUC, and calibration slope were estimated for both the final multivariable model and the combined SII–sdLDL-C model. A calibration slope close to 1.0 was considered indicative of satisfactory agreement between predicted and observed risks. All statistical tests were two-sided, and *P* < 0.05 was considered statistically significant.

## 3 Results

### 3.1 Comparison of baseline clinical and treatment characteristics

A total of 157 patients with UAP were included in this study, including 38 patients in the MACE group (24.20%) and 119 patients in the non-MACE group (75.80%). Among the 38 patients who experienced MACE, a total of 42 component events were recorded because four patients experienced two different components. Target vessel revascularization was the most frequent component, occurring in 20 patients (52.63%), followed by rehospitalization due to worsening heart failure in 11 patients (28.95%), non-fatal myocardial infarction in 8 patients (21.05%), and cardiac death in 3 patients (7.89%). Because the component events were not mutually exclusive, the percentages did not sum to 100%. The comparison of baseline clinical and treatment characteristics between the two groups is shown in Table 1. Compared with the non-MACE group, patients in the MACE group were older, had a higher proportion of diabetes, significantly higher Gensini scores, and a higher incidence of multi-lead ischemic changes (*P* < 0.05). There were no statistically significant differences between the two groups in sex distribution, BMI, hypertension, stroke, smoking history, previous PCI history, revascularization strategy during the index hospitalization, or the use of dual antiplatelet therapy, high-intensity statins, *β*-blockers, ACEI/ARB/ARNI, and nitrates at discharge (*P* > 0.05).

**Table 1:** Comparison of baseline clinical and treatment characteristics between the two groups.

Characteristic	MACE group (*n* = 38)	Non-MACE group (*n* = 119)	t/*χ*^2^	P
Sex	-	-	0.147	0.701
Male	24 (63.16%)	71 (59.66%)	-	-
Female	14 (36.84%)	48 (40.34%)	-	-
Age (years)	67.43 ± 9.18	61.82 ± 10.47	2.958	0.003
BMI (kg/m^2^)	24.83 ± 3.12	24.18 ± 3.37	1.053	0.293
Comorbidities	-	-	-	-
Hypertension	25 (65.79%)	69 (57.98%)	0.730	0.392
Diabetes mellitus	19 (50.00%)	34 (28.57%)	5.914	0.015
Stroke	6 (15.79%)	15 (12.61%)	0.252	0.615
Smoking history	-	-	0.451	0.501
Yes	18 (47.37%)	49 (41.18%)	-	-
No	20 (52.63%)	70 (58.82%)	-	-
Previous PCI history	-	-	0.614	0.432
Yes	11 (28.95%)	27 (22.69%)	-	-
No	27 (71.05%)	92 (77.31%)	-	-
Gensini score (points)	52.63 ± 18.47	36.88 ± 15.73	5.146	＜0.001
Multi-lead ischemic changes	-	-	9.284	0.002
Yes	23 (60.53%)	39 (32.77%)	-	-
No	15 (39.47%)	80 (67.23%)	-	-
Revascularization strategy during index hospitalization	—	—	0.161	0.923
Medical therapy alone	13 (34.21%)	45 (37.82%)	—	—
PCI	23 (60.53%)	68 (57.14%)	—	—
CABG	2 (5.26%)	6 (5.04%)	—	—
Dual antiplatelet therapy at discharge	—	—	—	0.728
Yes	35 (92.11%)	111 (93.28%)	—	—
No	3 (7.89%)	8 (6.72%)	—	—
Statin intensity at discharge	—	—	0.046	0.829
High intensity	25 (65.79%)	76 (63.87%)	—	—
Moderate or low intensity	13 (34.21%)	43 (36.13%)	—	—
*β*-blocker at discharge	—	—	0.062	0.803
Yes	27 (71.05%)	82 (68.91%)	—	—
No	11 (28.95%)	37 (31.09%)	—	—
ACEI/ARB/ARNI at discharge	—	—	0.040	0.841
Yes	24 (63.16%)	73 (61.34%)	—	—
No	14 (36.84%)	46 (38.66%)	—	—
Nitrates at discharge	—	—	0.054	0.817
Yes	26 (68.42%)	79 (66.39%)	—	—
No	12 (31.58%)	40 (33.61%)	—	—

PCI, percutaneous coronary intervention; CABG, coronary artery bypass grafting; ACEI, angiotensin-converting enzyme inhibitor; ARB, angiotensin receptor blocker; ARNI, angiotensin receptor–neprilysin inhibitor. Fisher's exact test was used for dual antiplatelet therapy at discharge.

### 3.2 Comparison of laboratory indicators

**Figure 2:**
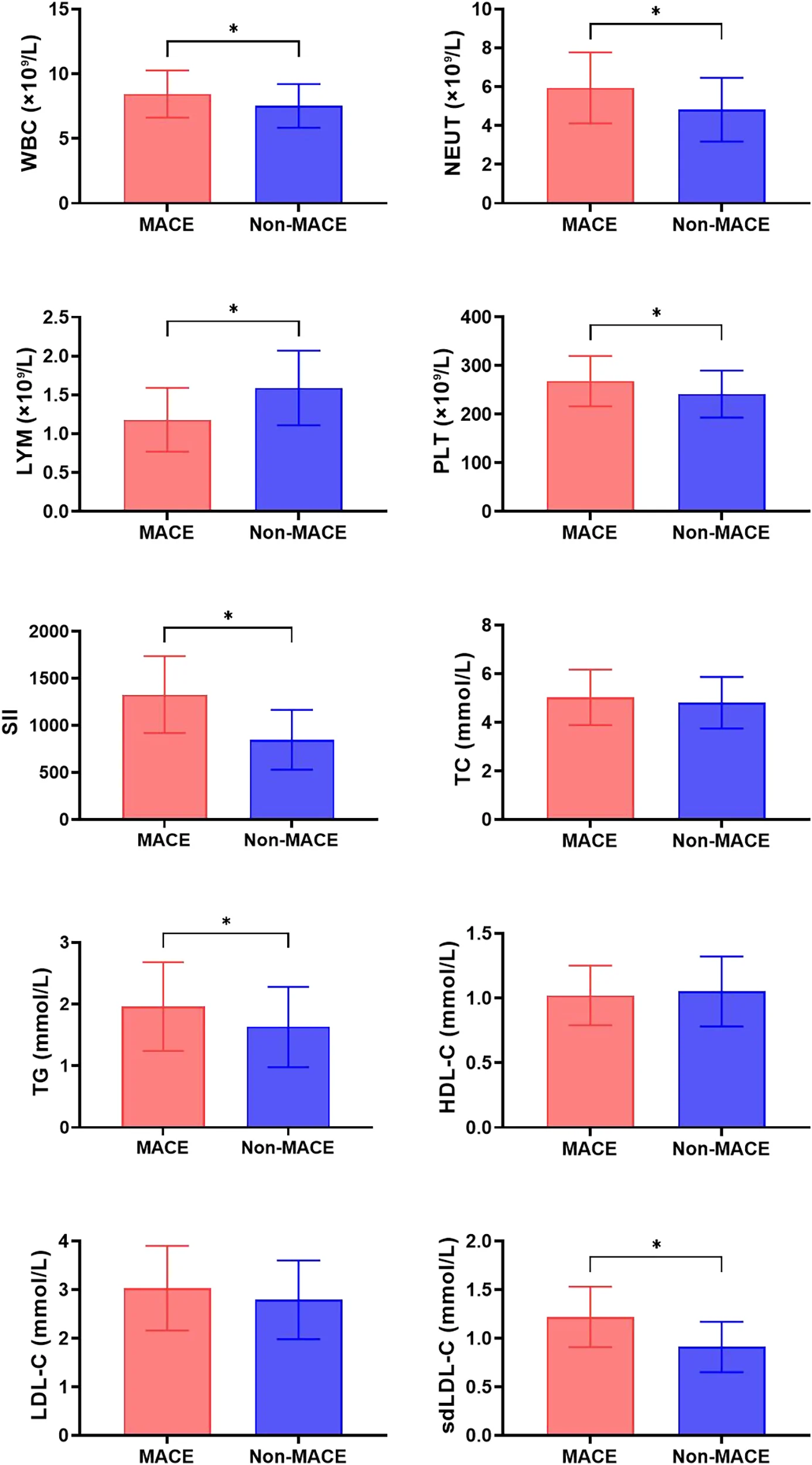
Comparison of laboratory indicators between the two groups. *indicates *P* < 0.05 for between-group comparisons.

The comparison of laboratory indicators between the two groups is shown in Table 2 and Figure 2. The levels of WBC, NEUT, PLT, SII, TG, and sdLDL-C in the MACE group were significantly higher than those in the non-MACE group, while the LYM level was significantly lower (all *P* < 0.05). There were no statistically significant differences in TC, HDL-C, or LDL-C between the two groups (*P* > 0.05).

**Table 2:** Comparison of laboratory indicators between the two groups.

Laboratory indicator	MACE group (*n* = 38)	Non-MACE group (*n* = 119)	t	P
WBC (×10⁹/L)	8.43 ± 1.82	7.52 ± 1.69	2.836	0.005
NEUT (×10⁹/L)	5.94 ± 1.83	4.82 ± 1.64	3.562	＜0.001
LYM (×10⁹/L)	1.18 ± 0.41	1.59 ± 0.48	4.739	＜0.001
PLT (×10⁹/L)	268.37 ± 51.84	241.62 ± 48.31	2.919	0.004
SII	1,326.48 ± 409.72	846.35 ± 318.56	7.522	＜0.001
TC (mmol/L)	5.03 ± 1.14	4.81 ± 1.06	1.093	0.275
TG (mmol/L)	1.96 ± 0.72	1.63 ± 0.65	2.653	0.008
HDL-C (mmol/L)	1.02 ± 0.23	1.05 ± 0.27	0.616	0.538
LDL-C (mmol/L)	3.03 ± 0.87	2.79 ± 0.81	1.561	0.120
sdLDL-C (mmol/L)	1.22 ± 0.31	0.91 ± 0.26	6.099	＜0.001

### 3.3 Multivariable logistic regression analysis

Whether MACE occurred was used as the dependent variable (No = 0; Yes=1). Seven variables with statistical significance in the univariate analyses—age, diabetes mellitus, TG, Gensini score, multi-lead ischemic changes, SII, and sdLDL-C—were entered into the multivariable logistic regression model according to the assignments shown in Table 3. Because 38 patients experienced MACE and seven candidate predictors were entered into the model, the event-per-variable ratio was approximately 5.4. This relatively limited ratio was considered when interpreting model stability and the potential risk of overfitting. The results showed that SII ≥1,100 (OR=3.58, 95%CI: 1.79–7.14, *P* < 0.001), sdLDL-C ≥ 1.02 mmol/L (OR = 2.91, 95%CI: 1.42–5.97, *P* = 0.003), Gensini score ≥45 points (OR = 2.67, 95%CI: 1.31–5.46, *P* = 0.006), and multi-lead ischemic changes on electrocardiography (OR = 2.43, 95%CI: 1.18–5.01, *P* = 0.017) were independently associated with MACE within 1 year in patients with UAP, whereas age, diabetes mellitus, and TG were not independently associated with MACE in the multivariable analysis (*P* > 0.05), as shown in Table 4. The Hosmer–Lemeshow test showed no evidence of poor model fit (*P* = 0.64).

**Table 3:** Variable assignment table.

Independent variable	Assignment method
Age	<65 years=0; ≥65 years=1
Diabetes mellitus	No = 0; Yes=1
TG	<1.70 mmol/L = 0; ≥1.70 mmol/L = 1
Gensini score	<45 points=0; ≥45 points=1
Multi-lead ischemic changes	No = 0; Yes=1
SII	<1,100 = 0; ≥1,100 = 1
sdLDL-C	<1.02 mmol/L = 0; ≥1.02 mmol/L = 1

**Table 4:** Multivariable logistic regression analysis of factors associated with the occurrence of MACE.

Factor	β	SE	Wald	P	OR	95%CI
Age ≥65 years	0.46	0.33	1.94	0.164	1.58	0.83–3.02
Diabetes mellitus	0.71	0.35	4.11	0.053	1.97	0.98–4.08
TG ≥1.70 mmol/L	0.38	0.31	1.50	0.221	1.46	0.80–2.68
Gensini ≥45 points	0.98	0.35	7.54	0.006	2.67	1.31–5.46
Multi-lead ischemic changes	0.89	0.37	5.74	0.017	2.43	1.18–5.01
SII ≥1,100	1.28	0.33	14.96	＜0.001	3.58	1.79–7.14
sdLDL-C ≥ 1.02 mmol/L	1.07	0.36	8.72	0.003	2.91	1.42–5.97

### 3.4 ROC curve analysis

**Figure 3:**
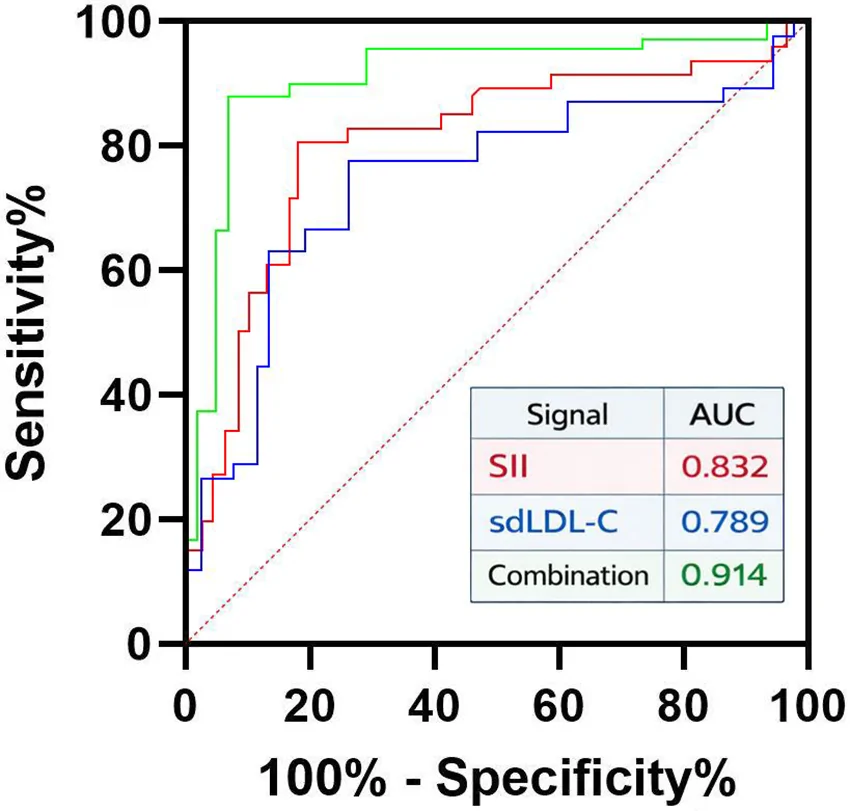
Receiver operating characteristic curves of SII, sdLDL-C, and the combined SII–sdLDL-C model for discriminating 1-year MACE.

The ROC curve analysis results are shown in Table 5 and Figure 3. The AUC of SII for discriminating 1-year MACE was 0.832 (95%CI: 0.760–0.904), with an optimal cutoff value of 1,100, a sensitivity of 78.95%, and a specificity of 74.79%. The AUC of sdLDL-C for discriminating 1-year MACE was 0.789 (95%CI: 0.702–0.876), with an optimal cutoff value of 1.02 mmol/L. The AUC of the combined model was 0.914 (95%CI: 0.867–0.961), and its discriminative performance was significantly better than that of either single indicator (DeLong test *P* < 0.05).

**Table 5:** Discrimiative performance of SII, sdLDL-C, and the combined SII–sdLDL-C model for 1-year MACE.

Indicator	Cutoff value	AUC	95% CI	Sensitivity (%)	Specificity (%)	Youden index
SII	1,100	0.832	0.760–0.904	78.95	74.79	0.537
sdLDL-C	1.02 mmol/L	0.789	0.702–0.876	73.68	70.59	0.442
Combined SII–sdLDL-C model	—	0.914	0.867–0.961	84.21	86.55	0.707

### 3.5 Internal validation of the prediction models

Internal validation was performed using 1,000 bootstrap resamples. The final multivariable logistic regression model had an apparent AUC of 0.926. The estimated bootstrap optimism was 0.028, resulting in an optimism-corrected AUC of 0.898. The calibration slope was 0.82, indicating some degree of overfitting.

For the combined SII–sdLDL-C model, the apparent AUC was 0.914. The estimated bootstrap optimism was 0.021, and the optimism-corrected AUC was 0.893. The calibration slope was 0.87, suggesting that the combined model retained good discriminative performance after internal validation, although mild overfitting remained. These findings indicate that the models retained relatively good discriminative performance but should be interpreted cautiously because of the limited number of patients who experienced MACE.

## 4 Discussion

This study evaluated the associations of SII and sdLDL-C with MACE within 1 year in patients with UAP from the complementary perspectives of inflammation and lipid metabolism. The results showed that patients who developed MACE presented with older age, a higher proportion of diabetes, a greater coronary artery lesion burden (higher Gensini score), and a higher incidence of multi-lead ischemic changes at baseline. Meanwhile, patients in the MACE group had higher WBC, NEUT, PLT, SII, TG, and sdLDL-C levels and lower LYM levels than those in the non-MACE group. More importantly, after adjustment for the measured demographic, metabolic, coronary lesion, and electrocardiographic variables included in the multivariable logistic regression model, SII ≥1,100 and sdLDL-C ≥ 1.02 mmol/L remained independently associated with MACE within 1 year. These findings indicate that SII and sdLDL-C provided information associated with 1-year MACE occurrence beyond the measured variables included in the model. However, because of the retrospective observational design, the results should not be interpreted as evidence that inflammation or sdLDL-related plaque vulnerability causally contributed to MACE. ROC curve analysis further showed that the combined SII–sdLDL-C model achieved a higher AUC than either marker alone, suggesting that the two indicators may provide complementary information for risk discrimination. Regarding the composition of the clinical endpoint, target vessel revascularization was the most frequent event, followed by rehospitalization due to worsening heart failure, non-fatal myocardial infarction, and cardiac death. This distribution indicates that the composite endpoint in the present cohort was mainly composed of non-fatal events. However, because the number of each component was limited and some patients experienced more than one component, the component data were reported descriptively and were not used for separate regression or ROC analyses.

Inflammation is increasingly recognized as an important component of the pathophysiology and clinical outcomes of UAP. Atherosclerosis is therefore regarded not only as a process of lipid deposition but also as a persistent immune-inflammatory response. Neutrophils can promote endothelial injury and plaque vulnerability by releasing reactive oxygen species, proteolytic enzymes, and forming neutrophil extracellular traps (NETs), while also enhancing the tendency for thrombosis formation (15). Platelets not only participate in the coagulation cascade but also interact with inflammatory cells to promote adhesion, chemotaxis, and amplification of local inflammatory responses (16). In contrast, decreased lymphocyte levels often indicate enhanced stress responses, impaired immune regulation, and an unfavorable inflammatory balance in the body (17). SII integrates neutrophils, platelets, and lymphocytes into a single index, making it easier to capture the pathological state characterized by the coexistence of “pro-inflammatory, pro-thrombotic, and immunosuppressive” conditions (18). In this study, SII was significantly higher in the MACE group and remained independently associated with MACE in the multivariable analysis. This finding supports the potential value of SII as an integrated inflammatory marker for risk assessment; however, the present study did not directly evaluate inflammatory pathways, thrombogenic activity, or plaque instability. This finding is consistent with previous studies (19, 20) that used SII for risk stratification of cardiovascular events. Compared with individual inflammatory markers, SII integrates information from several inflammatory and hematologic components and may provide a broader assessment of systemic inflammatory status. Nevertheless, its underlying biological role was not directly examined in the present study.

In parallel with the inflammatory pathway reflected by SII, sdLDL-C provides information on atherogenic lipoprotein subfractions. Compared with conventional LDL-C, sdLDL particles are smaller and denser, making them more likely to penetrate the vascular endothelium and remain in the vascular wall. They are also more susceptible to oxidative modification, forming oxidized LDL and other highly pro-inflammatory substances that induce monocyte–macrophage uptake and transformation into foam cells, thereby promoting plaque growth (21, 22). In addition, sdLDL has been associated with endothelial dysfunction, smooth muscle cell migration, and decreased stability of the fibrous cap, making it more closely related to plaque vulnerability and rupture risk (23, 24). In the present study, sdLDL-C was significantly higher in the MACE group and remained independently associated with MACE in the multivariable analysis. This association is consistent with the atherogenic characteristics of sdLDL described in previous studies, but the present data do not establish that sdLDL-C directly promoted plaque destabilization or subsequent cardiovascular events. In clinical practice, conventional lipid profiles (TC, LDL-C, HDL-C, TG) are easily influenced by short-term physiological conditions, therapeutic interventions, and timing of measurement. As a lipid subfraction marker, sdLDL-C may help identify residual lipid-related risk among patients with similar LDL-C concentrations. This may explain why in some studies sdLDL-C has shown closer associations with acute cardiovascular events or long-term adverse outcomes compared with traditional LDL-C (25, 26). Another noteworthy finding was that TG was higher in the MACE group in the univariable analysis but did not remain independently associated with MACE in the multivariable model. Such findings are not uncommon in studies of lipid metabolism and prognosis. Possible explanations include: (1) TG is often associated with metabolic syndrome, insulin resistance, and the formation of sdLDL, and may therefore influence risk indirectly by promoting increases in sdLDL levels. Once sdLDL-C is included in the model, the independent contribution of TG may be weakened; (2) the sample size and number of events limit the number of variables that can be included in the model. Some indicators, although associated with outcomes, may lose statistical significance after multivariate adjustment due to smaller effect sizes or collinearity with other variables. Similarly, LDL-C has shown inconsistent predictive performance for short- to medium-term MACE in some studies (27, 28). On the one hand, widespread use of lipid-lowering therapy has led to more homogeneous LDL-C levels among patients; on the other hand, differences in LDL particle characteristics (such as the proportion of sdLDL) may be masked by the total LDL-C concentration. Therefore, emphasizing sdLDL-C rather than LDL-C alone may improve the identification of risk heterogeneity to some extent.

In addition to inflammation and lipid metabolism, coronary lesion burden and the degree of myocardial ischemia remain important clinical dimensions in the prognostic assessment of UAP. In this study, higher Gensini scores and multi-lead ischemic changes were more common in the MACE group and remained independently associated with 1-year MACE in the multivariable analysis, indicating that both anatomical lesion burden and electrocardiographic ischemic manifestations provided relevant prognostic information. The Gensini score quantifies the severity of coronary artery stenosis and lesion distribution, representing the “extent and severity of disease” (29). Multi-lead ischemic changes reflect the extent or severity of myocardial ischemia and often indicate a larger area of myocardial perfusion impairment or a more unstable supply–demand balance (30). Taken together, the findings from the full multivariable analysis support a multidimensional assessment encompassing lesion burden, ischemic manifestations, inflammatory status, and lipid-related risk.

The relatively high apparent AUC of the combined model was an important finding of this study. However, after bootstrap correction, the AUC decreased from 0.914 to 0.893, indicating that part of the apparent predictive performance may have resulted from optimism related to model development and evaluation in the same cohort. Therefore, the combined model should currently be regarded as an exploratory risk-stratification tool rather than a fully validated clinical prediction model. SII and sdLDL-C represent different clinical domains, namely systemic inflammatory and hematologic status and lipid subfraction characteristics. Their combined use may therefore provide complementary statistical information for discrimination. This interpretation is biologically plausible; however, the underlying mechanisms were not directly tested in the present study. Patients may differ in the relative contributions of inflammatory burden and lipid subfraction abnormalities, which may partly explain the complementary information provided by the two markers. Using two indicators reflecting different biological domains may provide more comprehensive risk information than using either indicator alone. Nevertheless, whether the combined model improves the identification and clinical management of high-risk patients requires confirmation through external validation and prospective impact studies. The cutoff values of 1,100 for SII and 1.02 mmol/L for sdLDL-C were derived from the present cohort and should therefore be regarded as exploratory rather than universal clinical decision thresholds. The direction of the observed associations is consistent with previous studies linking higher SII and sdLDL-C levels to adverse cardiovascular outcomes (19, 20, 24–26). However, direct comparison of exact cutoff values is limited by differences in study populations, outcome definitions, follow-up periods, and sdLDL-C assay methods, reagent systems, and analytical platforms. Therefore, these thresholds may provide supplementary information for identifying patients who warrant closer clinical assessment, but they should not replace established risk assessment methods or independently determine treatment decisions until validated in external cohorts. From the perspective of risk stratification strategies, the value of the combined model does not lie in replacing existing scoring systems but rather in providing a simple, accessible, and relatively low-cost supplementary tool to help clinicians further identify patients with UAP who may require more intensive short- to medium-term intervention or closer follow-up.

Overall, this study found that SII and sdLDL-C were independently associated with MACE within 1 year and that their combination improved discrimination in the present cohort. These findings support further evaluation of the two markers as supplementary components of risk assessment. However, whether their use improves clinical decision-making, follow-up strategies, treatment adherence, or patient outcomes cannot be determined from this retrospective study and requires prospective impact studies.

## 5 Clinical implications and limitations

This study has potential clinical implications. First, SII is derived from a combination of three routine blood test parameters, while sdLDL-C testing has become available in an increasing number of hospitals. Both indicators are clinically accessible, and their measurement during hospitalization may provide supplementary information for risk assessment in patients with UAP. Second, the combined SII–sdLDL-C model showed better discriminative ability than either marker alone and may provide supplementary information for risk assessment in patients with UAP. However, the cohort-derived thresholds should not be used as stand-alone criteria for determining treatment intensity or follow-up strategies before external validation. In addition, the full multivariable analysis considered coronary lesion burden and ischemic manifestations, emphasizing the importance of multidimensional evaluation in clinical practice. However, several limitations should be noted. (1) This was a single-center retrospective study, which may involve selection and information bias and limit the generalizability of the findings. Because the exposures and outcomes were retrospectively observed rather than experimentally assigned, causal relationships and underlying biological mechanisms cannot be established. Revascularization strategy during the index hospitalization and major discharge medications were available from the medical records and were comparable between the MACE and non-MACE groups. However, long-term medication adherence, subsequent treatment changes, dose adjustments, and cumulative medication exposure during follow-up could not be consistently obtained. Therefore, residual confounding related to post-discharge treatment and adherence cannot be excluded. (2) Only 38 patients experienced at least one MACE during follow-up. Seven candidate predictors were evaluated in the initial multivariable analysis, corresponding to approximately 5.4 events per candidate variable. Therefore, model instability and overfitting could not be completely excluded. Internal validation using 1,000 bootstrap resamples showed that the AUC of the final multivariable model decreased from 0.926 to an optimism-corrected value of 0.898, with a calibration slope of 0.82. The AUC of the combined SII–sdLDL-C model decreased from 0.914 to 0.893, with a calibration slope of 0.87. These findings indicate that the models retained relatively good discrimination but showed mild optimism. Moreover, the cutoff values were derived and evaluated in the same cohort, which may have resulted in information loss and optimistic performance estimates. No independent external cohort was available in this single-center study. Therefore, although bootstrap validation provided an estimate of internal optimism, the generalizability of the models and cutoff values remains unverified, and external validation is required before clinical application. (3) Although sdLDL-C was measured in a single central laboratory using a standardized direct assay and routine quality-control procedures, this retrospective study relied on a single baseline measurement for each patient and did not specifically evaluate within-person repeatability or study-specific between-run analytical variation. In addition, sdLDL-C results may vary across assay principles, reagent systems, analytical platforms, and institutional reference intervals. Therefore, the cutoff value of 1.02 mmol/L identified in this cohort should be interpreted in the context of the assay used in the present study and requires verification using other analytical platforms and in independent external cohorts. (4) The follow-up period was limited to 1 year, which may not reflect longer-term outcomes. In addition, because the exact date of the first MACE was not consistently available for all patients, the endpoint was analyzed as a fixed 1-year binary outcome, and time-to-event methods such as Cox regression could not be reliably applied. Therefore, the present findings reflect associations with the occurrence of MACE within 1 year rather than differences in the timing of events. (5) Although the distribution of individual MACE components was reported descriptively, the number of cardiac deaths, non-fatal myocardial infarctions, target vessel revascularizations, and heart failure hospitalizations was limited. In addition, some patients experienced more than one component event. Therefore, reliable component-specific regression and ROC analyses could not be performed. Larger studies are needed to determine whether SII and sdLDL-C have different associations with individual cardiovascular outcomes.

## 6 Conclusion

In patients with UAP, elevated SII and sdLDL-C levels were independently associated with MACE within 1 year in the present cohort, and their combined model showed better discrimination than either marker alone. These findings reflect statistical associations and discriminative performance and do not establish causal effects or biological mechanisms. Because the analysis was based on a fixed 1-year binary endpoint and did not account for the timing of events, the findings should not be interpreted as a time-to-event prognostic assessment. After 1,000 bootstrap resamples, the optimism-corrected AUC of the combined model was 0.893, with a calibration slope of 0.87, indicating relatively good internal discrimination but mild overfitting. SII and sdLDL-C may therefore warrant further evaluation as supplementary markers for risk stratification rather than replacements for established clinical assessment methods. Given the limited number of patients who experienced MACE, the data-derived cutoff values, residual confounding, and lack of external validation, these findings should be regarded as exploratory and require confirmation in larger, independent, multicenter, and prospective cohorts.

## Data Availability

The original contributions presented in the study are included in the article/Supplementary Material, further inquiries can be directed to the corresponding author.
